# Possible involvement of neuropeptide and neurotransmitter receptors in Adenomyosis

**DOI:** 10.1186/s12958-021-00711-6

**Published:** 2021-02-19

**Authors:** Xiaofang Xu, Xianjun Cai, Xishi Liu, Sun-Wei Guo

**Affiliations:** 1 Department of Obstetrics and Gynecology, Ningbo No. 7 Hospital, Ningbo, Zhejiang, 315200 China; 2 Shanghai Obstetrics and Gynecology Hospital, Fudan University, 419 Fangxie Road, Shanghai, 200011 China; 3 Shanghai Key Laboratory of Female Reproductive Endocrine-Related Diseases, Fudan University, Shanghai, China

**Keywords:** Adenomyosis, Adrenergic receptor β2, α7 nicotinic acetylcholine receptor, Calcitonin gene related-protein receptors, Neurokinin receptor 1, Receptor activity modifying protein 1

## Abstract

**Background:**

Accumulating data indicate that sensory nerve derived neuropeptides such as substance P and calcitonin gene related-protein (CGRP) can accelerate the progression of endometriosis via their respective receptors, so can agonists to their respective receptors receptor 1 (NK1R), receptor activity modifying protein 1 (RAMP-1) and calcitonin receptor-like receptor (CRLR). Adrenergic β2 receptor (ADRB2) agonists also can facilitate lesional progression. In contrast, women with endometriosis appear to have depressed vagal activity, concordant with reduced expression of α7 nicotinic acetylcholine receptor (α7nAChR). The roles of these receptors in adenomyosis are completely unknown.

**Methods:**

Adenomyotic tissue samples from 30 women with adenomyosis and control endometrial tissue samples from 24 women without adenomyosis were collected and subjected to immunohistochemistry analysis of RAMP1, CRLR, NK1R, ADRB2 and α7nAChR, along with their demographic and clinical information. The extent of tissue fibrosis was evaluated by Masson trichrome staining.

**Results:**

We found that the staining levels of NK1R, CRLR, RAMP1 and ADRB2 were all significantly elevated in adenomyotic lesions as compared with control endometrium. In contrast, α7nAChR staining levels were significantly reduced. The severity of dysmenorrhea correlated positively with lesional ADRB2 staining levels.

**Conclusions:**

Our results suggest that SP, CGRP and noradrenaline may promote, while acetylcholine may stall, the progression of adenomyosis through their respective receptors on adenomyotic lesions. Additionally, through the activation of the hypothalamic-pituitary-adrenal (HPA)-sympatho-adrenal-medullary (SAM) axes and the lesional overexpression of ADRB2, adenomyosis-associated dysmenorrhea and adenomyotic lesions may be mutually promotional, forming a viscous feed-forward cycle.

**Supplementary Information:**

The online version contains supplementary material available at 10.1186/s12958-021-00711-6.

## Introduction

Adenomyosis is a prevalent, benign gynecological condition characterized by infiltration of endometrial tissues into the myometrium [1]. Its presenting symptoms include a soft and diffusely enlarged uterus with pelvic pain, abnormal uterine bleeding (AUB), and subfertility [2–5], but its pathogenesis and pathophysiology are poorly understood [6–8]. While approximately one third of adenomyotic cases are asymptomatic [9], dysmenorrhea is the most prevalent symptom besides AUB [10].

Evidence accumulated in the last few years indicates that adenomyotic lesions, just like endometriotic lesions [11–13], are wounds undergoing repeated tissue injury and repair (ReTIAR) owing to cyclic bleeding of ectopic endometrium [14, 15]. It is well documented that neuromediators secreted by sensory and autonomic nerves are actively involved in all phases of tissue repair [16]. For example, neutrophins such as nerve growth factor (NGF) and their receptors are implicated in tissue repair and remodeling [17]. The neuropeptide substance P (SP) released by sensory nerves on the wounding site induces vasodilatation and vascular permeability that promote plasma extravasation [18, 19] via its receptor, neurokinin receptor 1 (NK1R) [20, 21]. Sensory nerve derived calcitonin gene related-protein (CGRP) also is implicated in vasodilatation and inflammation [22]. Both SP and CGRP can modulate collagen production during skin wound healing [23]. SP is known to accelerate the normal acute and chronic wound healing processes [24–26], while sensory denervation impairs cutaneous wound healing [27–29]. Similarly, sympathetic denervation by oxidopamine also leads to impaired wound healing which was associated with a decrease of neurogenic inflammation [30, 31].

As wounds, endometriotic lesions are highly vascularized, at least initially [32], and richly innervated [33, 34]. In fact, increased nerve fiber density is a notable feature of the lesional microenvironment, especially in deep endometriosis [35–38]. Similarly, adenomyotic lesions also are highly vascularized [14, 39, 40]. However, whether adenomyotic lesions are similarly innervated appears to be controversial [41–43]. Adenomyotic lesions also seem to have impaired sympathetic innervation [42].

We have previously reported that sensory nerve derived SP and CGRP can accelerate the progression of endometriosis via their respective receptors [44, 45], so can β2 adrenergic receptor (ADRB2) agonists [46] and an NK1R agonist [44]. More remarkably, we recently reported that lesional expression of ADRB2 correlated positively with the severity of dysmenorrhea in women with endometriosis, suggesting a positive feed-forward loop between pain and lesional progression [47]. These results clearly demonstrate that the promotional roles of neuropeptides, such as SP and CGRP, and neurotransmitters, such as adrenaline and noradrenaline, which are derived from sensory and sympathetic nerves, respectively, in the progression of endometriosis via their respective receptors.

One important nerve system that has not been investigated at all in adenomyosis is the vegus nerves. Women with endometriosis appear to have depressed vagal activity [48]. Vagotomy, which mimics reduced vagal activity, has recently been reported to accelerate the progression of endometriosis but vegus nerve stimulation substantially stalls the lesion progression in mouse [48].

In retrospect, this is not surprising, since in the last two decades it has been recognized that there is a complex and intricate crosstalk between the nervous and immune systems through a plethora of cytokines, neurotransmitters and hormones, which serves as counter-regulatory mechanisms capable of dampening inflammation and restoration of homeostasis [49, 50]. In fact, within the framework of cholinergic anti-inflammatory pathway [51], the vegus nerves are known play a pivotal role in anti-inflammation through the activation of the α7 nicotinic acetylcholine receptor (α7nAChR) [52, 53]. Incidentally, treatment with an α7nAChR agonist has been reported to suppress the formation of endometriotic lesions [54]. However, whether α7nAChR is expressed in endometriosis or adenomyosis is unclear.

In light of these findings, it is plausible that the neuropeptides and neurotransmitters may act on ectopic endometrium through their respective receptors. In this study, we hypothesized that adenomyotic lesions have increased immunoreactivity against NK1R, ADRB2, and CGRP receptors CRLR and RAMP1 but decreased immunoreactivity against α7nAChR. Since fibrosis is a notable feature of adenomyosis and a rough proxy for lesional progression [14, 55], we also evaluated the extent of lesion fibrosis to see whether it has any relationship with these receptors.

## Materials and methods

This study was approved by the Institutional Ethics Review Board of the Shanghai OB/GYN Hospital. All tissue samples were obtained after written, full and informed consent from recruited subjects.

### Human samples

Adenomyotic tissue samples were obtained, after informed written consent, during hysterectomy from premenopausal patients with ultrasonically, laparoscopically and histologically diagnosed adenomyosis (*n* = 30), who were admitted to the Shanghai OB/GYN Hospital, Fudan University, from January, 2018 to December, 2019. For controls, we also collected, after informed consent, endometrial tissue samples through curettage from 24 women with cervical intraepithelial neoplasia (CIN-III) but free of endometriosis, adenomyosis, and uterine fibroids, who were age- and menstrual phase-matched (in frequency) with patients with adenomyosis. The selection of the controls was based solely on age and menstrual phase besides disease status. None of the recruited subjects had any malignancy or other inflammatory disease, or received any hormonal or anti-platelet treatment for at least 3 months prior to the recruitment. Information on uterine size, severity of dysmenorrhea, amount of menses was also recorded.

### Immunohistochemistry (IHC) analysis

Tissue samples were fixed with 10% formalin (w/v) and paraffin embedded. Serial 4-μm sections were obtained from each block, with the first resultant slide being stained with hematoxylin and eosin to confirm pathologic diagnosis [56], and the subsequent slides for IHC analysis for the two receptors of CGRP, i.e. receptor activity modifying protein 1 (RAMP-1) (1:100, ab203282, Abcam, Cambridge, UK), calcitonin receptor-like receptor (CRLR) (1:50, ab84467, Abcam), SP receptor NK1R (1:100, NB300–119, Novus, CO, USA), noradrenaline receptor ADRB2 (1:200, ab182136, Abcam), nicotinic acetylcholine receptor α7nAChR (1:200, ab10096, Abcam).

Routine deparaffinization and rehydration procedures were performed, as reported previously [44, 56]. For antigen retrieval, the slides were heated at 98 °C in a citrate buffer (pH 6.0) for a total of 30 min and then cooled to room temperature. The slides were then incubated with the primary antibodies overnight at 4 °C. After the slides were rinsed with PBS, the horse radish peroxidase-labeled secondary antibody Detection Reagent (Sunpoly-HII; BioSun Technology Co, Ltd., Shanghai, China) was added and incubated at room temperature for 30 min. The bound antibody complexes were stained with diaminobenzidine for 3 to 5 min or until appropriate for microscopic examination and then counterstained with hematoxylin for 30 s and mounted. Images were obtained with the microscope (Olympus BX53; Olympus, Tokyo, Japan) fitted with a digital camera (Olympus DP73; Olympus). For each marker from every patient, 3 slides were used, and 5 randomly selected images at 400× magnification of each slide were taken to get a mean optional density value by Image Pro-Plus 6.0.

Mouse brain (for RAMP1, CRLR, NK1R, and ADRB2) and mouse kidney (for α7nAChR) tissue samples were used as positive controls. For negative controls, mouse brain (for RAMP1, CRLR, NK1R, and ADRB2) and human endometrial (for α7nAChR) tissue samples were incubated with rabbit or mouse serum instead of primary antibodies (Supplementary Information Fig. S1).

### Masson trichrome staining

Masson trichrome staining was used for the evaluation and semi-quantification of collagen fibers in endometriotic tissue samples. Tissue sections were deparaffinized in xylene and rehydrated in a graded alcohol series, then were immersed in Bouin’s solution at 37 °C for 2 h, which was made with saturated 75 mL of picric acid, 25 mL of 10% (w/v) formalin solution and 5 mL of acetic acid. Sections were stained using the Masson’s Trichrome Staining kit (Baso, Wuhan, China) following the manufacturer’s instructions. The proportion of the areas of the collagen fiber layer stained in blue relative to the entire field of the ectopic endometrium, calculated by the Image Pro-Plus 6.0, was taken as the percentage of fibrotic content in the tissues or lesions.

### Statistical analysis

The comparison of distributions of continuous variables between two groups was made using the Wilcoxon’s test. Multivariate linear regression analyses were used to determine whether age, menstrual phase, parity, presence or absence of uterine fibroids, presence of absence of deep endometriosis, and group identity (adenomyosis or control) were associated with immunostaining levels of different markers in human samples. Pearson’s correlation coefficient was used to gauge the correlation between to continuous variables, and Spearman’s rank correlation coefficient was used to evaluate the correlation between one continuous variable and one ordinal variable. The normality assumption was checked by plotting the regression residuals using Q-Q plot. *P*-values of < 0.05 were considered statistically significant. All computations were made with R 4.0.2 [57] (www.r-project.org).

## Results

The characteristics of the recruited patients with adenomyosis and the control subjects are listed in Table 1. As shown in the table, the two groups of patients were comparable in age and menstrual phase. However, patients with adenomyosis had significantly lower parity, heavier menses and more severe dysmenorrhea.

**Table 1:** Characteristics of recruited patients with and without adenomyosis

Variable name	Control (***n*** = 24)	Adenomyosis (***n*** = 30)	***p***-value
Age			
*Mean (±S.D.)*	41.3 ± 4.1	40.0 ± 2.2	0.13
*Median (range)*	42 (31—47)	40 (34—43)	
Parity			
*0*	0 (0.0%)	4 (13.3%)	0.23
*1*	14 (58.3%)	16 (53.3%)	
*≥ 2*	10 (41.7%)	10 (33.3%)	
Menstrual phase			
*Proliferative*	14 (58.3%)	16 (53.3%)	0.79
*Secretory*	10 (41.7%)	14 (46.7%)	
Amount of menses			
*Light*	0 (0.0%)	1 (3.3%)	1.4 × 10^−5^
*Moderate*	24 (100.0%)	14 (46.7%)	
*Heavy*	0 (0.0%)	15 (50.0%)	
Severity of dysmenorrhea			
*None*	22 (91.6%)	3 (10.0%)	4.8 × 10^−10^
*Mild*	1 (4.2%)	3 (10.0%)	
*Moderate*	1 (4.2%)	6 (20.0%)	
*Severe*	0 (0.0%)	18 (60.0%)	
Ovarian endometrioma			
*Absent*	24 (100.0%)	28 (93.3%)	0.50
*Present*	0 (0.0%)	2 (6.7%)	
Deep endometriosis			
*Absent*	24 (100.0%)	25 (83.3%)	0.059
*Present*	0 (0.0%)	5 (16.7%)	
Uterine fibroids			
*Absent*	24 (100.0%)	21 (70.0%)	0.0029
*Present*	0 (0.0%)	9 (30.0%)	
Uterine size (in cm^3^)			
*Mean (±S.D.)*	63.3 ± 21.0	304.8 ± 103.8	1.4 × 10^−15^
*Median (range)*	63.3 (33.0—116.8)	295.9 (130.3—531.1)	

We carried out immunohistochemistry analysis for CGRP receptors RAMP1 and CRLR, SP receptor NK1R, adrenergic receptor ADRB2, and α7nChR for adenomyotic lesions, as well as the extent of lesion fibrosis by Masson trichrome staining.

**Fig. 1:**
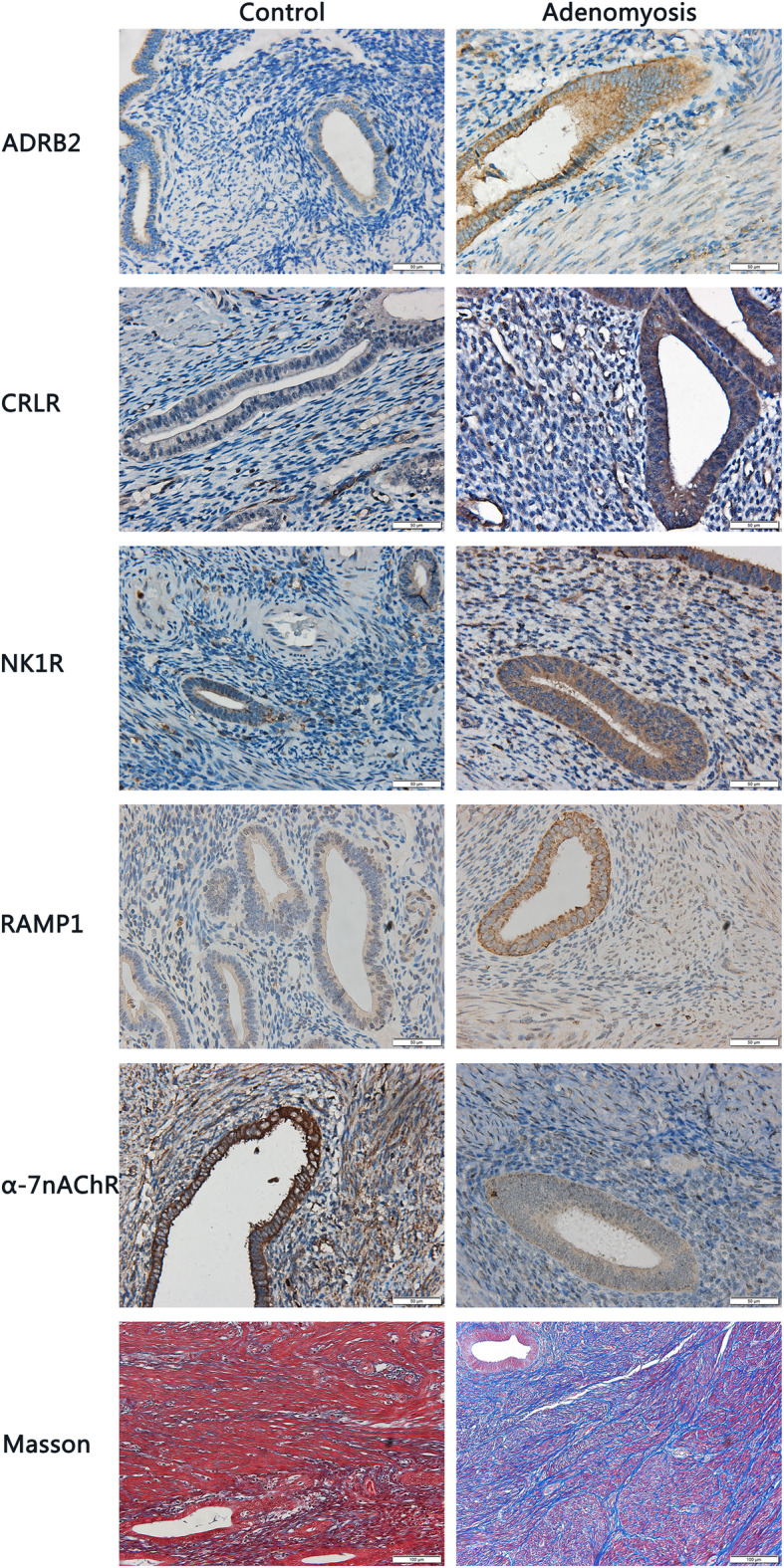
Representative photomicrographs of immunohistochemistry analysis of calcitonin receptor like receptor (CRLR), receptor activity modifying protein 1 (RAMP-1), neurokinin 1 receptor (NK1R), adrenergic receptor β2 (ADRB2), and α7 nicotinic acetylcholine receptor (α7nAChR) in the epithelial and stromal components in control) and adenomyotic tissues. Magnification: × 400. Scale bar = 50 μm

We found that RAMP-1, CRLR, NK1R, ADRB2 and α7nAChR staining was seen in both epithelial and stromal cells of adenomyotic lesions and control endometrial tissues, The staining of RAMP-1, CRLR, NK1R and α7nAChR was seen in both epithelial and stromal cells and localized in the cytomembrane. The ADRB2 immunoreactivity was seen in both epithelial and stromal cells and was localized in the cytoplasm (Fig. 1). Overall, the staining of RAMP-1, CRLR, NK1R, and ADRB2 in adenomyotic lesions was much intense than control endometrium. In contrast, the staining of α7nAChR was much weaker in adenomyotic lesions than control endometrium (Fig. 1).

**Fig. 2:**
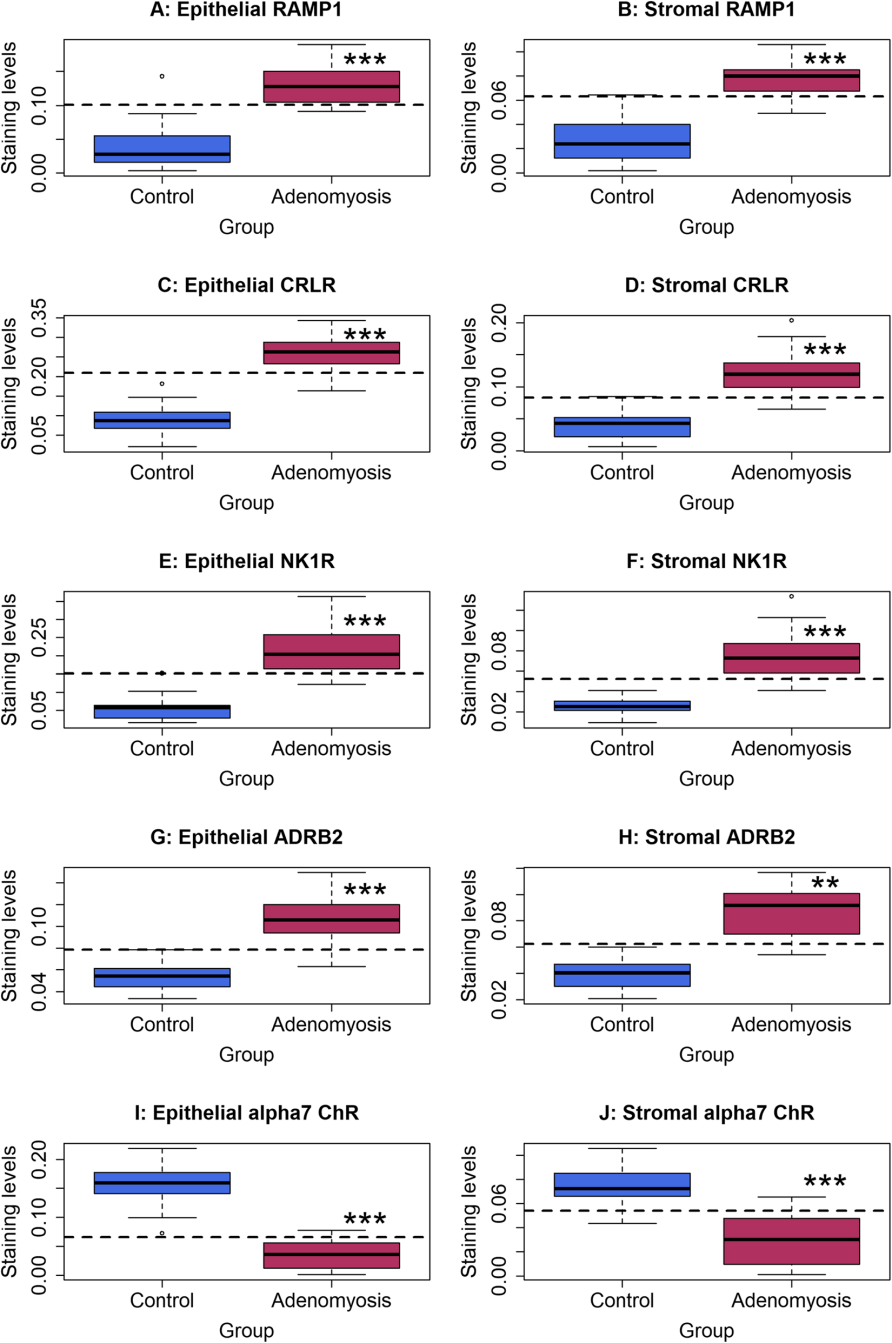
Summary of immunohistochemistry analyses by boxplots. Epithelial **a** and stromal **b** staining of RAMP1, epithelial **c** and stromal **d** staining of CRLR, epithelial **e** and stromal **f** staining of NK1R, epithelial **g** and stromal **h** staining of ADRB2, and epithelial **i** and stromal **j** staining of α7nAChR. In all figures, the dashed line represents the regression line. The comparison was made between patients with adenomyosis and controls (Wilcoxon’s rank test). Symbols for statistical significance levels: **: *p* < 0.01; ***: *p* < 0.001

We found that the staining levels of RAMP1, CRLR, NK1R, and ADRB2 were significantly elevated in both the epithelial and stromal components in adenomyotic lesions as compared with control endometrium (all *p*-values < 3.0 × 10^− 9^; Fig. 2a-h). In contrast, the staining levels of α7nAChR were significantly reduced in both compared with control endometrium as compared with the control endometrium (both p-values < 7.1 × 10^− 10^; Fig. 2i, j). For all markers, the staining levels in the epithelial and stromal components were highly positively correlated (all r ≥ 0.82, all *p* < 3.7 × 10^− 14^). Multiple linear regression incorporating age, menstrual phase, parity, presence or absence of uterine fibroids, presence or absence of deep endometriosis, presence or absence of ovarian endometriomas, and group identity (adenomyosis or control) confirmed that adenomyotic lesions were associated with higher staining of RAMP1, CRLR, NK1R, ADRB2 but lower staining of α7nAChR in both epithelial and stromal components (all *p*-values < 1.2 × 10^− 12^, all *R*^*2*^’s ≥ 0.63).

**Fig. 3:**
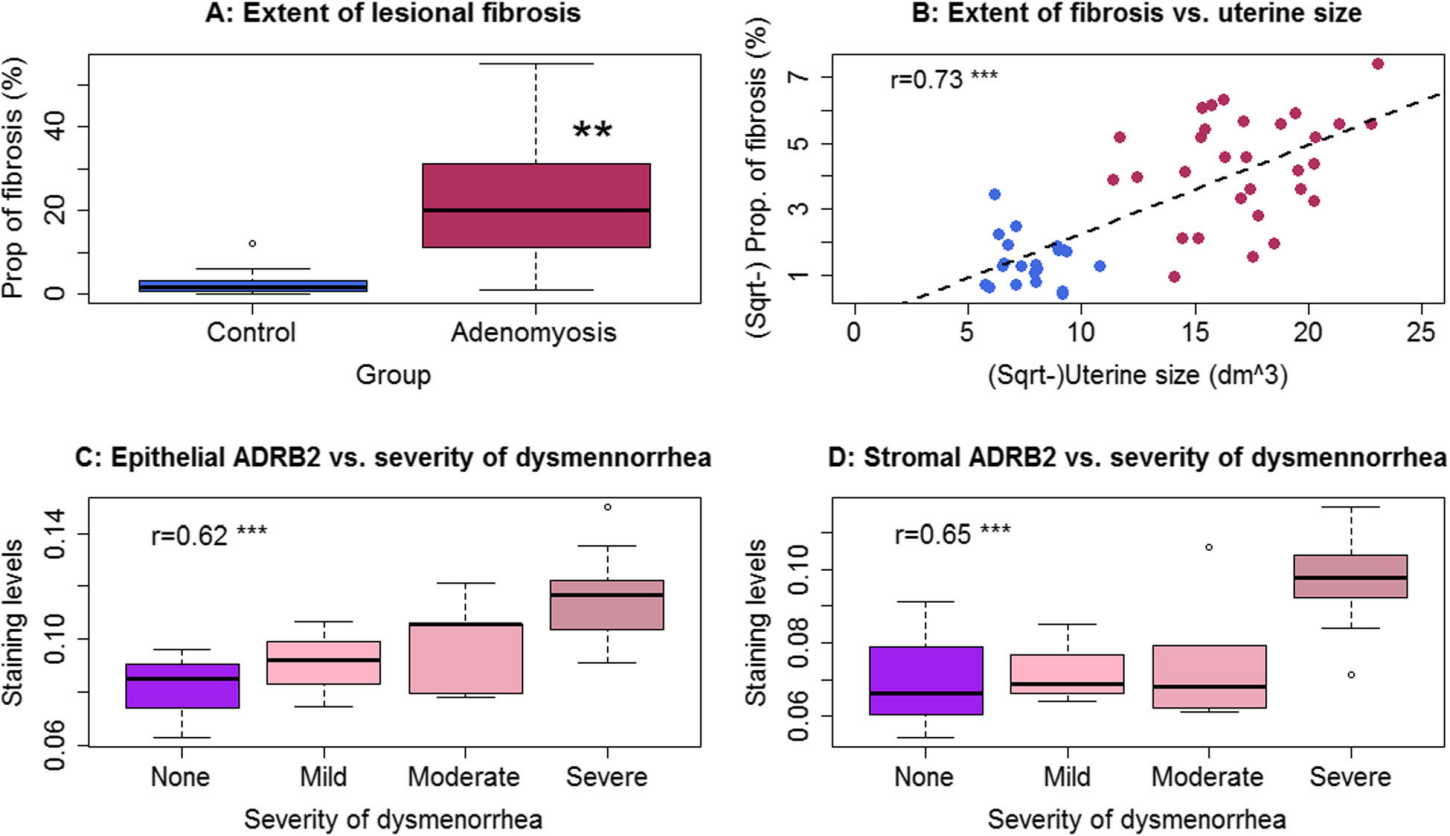
**a** Boxplot showing the summary results of the extent of fibrosis in adenomyotic lesions and control endometrium (Wilcoxon’s test). **b** Scatter plot showing the relationship between the extent of lesional fibrosis and uterine size (in cm^3^). Data from the women with adenomyosis (in maroon color) and control patients (in royal blue color) are represented in different colors. Pearson’s correlation coefficient, along with its statistical significance level, is also shown. The dashed line represents the regression line. **c** and **d** Boxplots showing, respectively, the difference in epithelial and stromal ADRB2 in women with adenomyosis complaining different severity of dysmenorrhea. For panels **c** and **d**, the Spearman’s correlation coefficient and its statistical significance level are shown. Symbols for statistical significance levels: **: *p* < 0.01; ***: *p* < 0.001

Consistent with previously reported [14], adenomyotic lesions had significantly higher extent of fibrosis than control endometrium (*p* = 1.4 × 10^− 9^; Fig. 3a). Multiple linear regression incorporating age, menstrual phase, parity, presence or absence of uterine fibroids, presence or absence of deep endometriosis, presence or absence of ovarian endometriomas, and group identity (adenomyosis or control) confirmed that adenomyotic lesions were associated with higher fibrotic content (*p* = 1.6 × 10^− 9^, *R*^*2*^ = 0.58). In addition, the uterine size correlated positively with the extent of fibrosis (r = 0.73, *p* = 1.2 × 10^− 9^; Fig. 3b). While no relationship between lesional staining levels and the amount of menses (all *p*-values > 0.21), the lesional staining levels of ADRB2 in both epithelial and stromal components correlated positively with the severity of dysmenorrhea (Spearman’s r = 0.62, *p* = 0.0004, and r = 0.65, *p* = 0.0002, respectively; Fig. 3c, d). In both epithelial and stromal components, we found that the staining levels of α7nAChR correlated negatively with that of NK1R, CRLR, RAMP1, and ADRB2 (all r’s < − 0.68, all p’s < 8.9 × 10^− 8^).

## Discussion

In adenomyosis, the role of nervous systems has been traditionally investigated in the context of pain, in that the hyperinnervation within or surrounding adenomyotic lesions is often taken as evidence to support the notion of increased nociception, nociceptor activation and thus enhanced perception of pain. Indeed, it has been reported that the PGP9.5-positive nerve fiber density in the basal layer of the endometrium or myometrium is significantly increased in women with adenomyosis complaining pain, and that neurofilament (NF)-positive, but not PGP9.5-positive, nerve fibers are found in the basal layer of the endometrium and myometrium in women with adenomyosis [41]. While Mechsner and her associates also found that adenomyotic lesions are not innervated [42], a more recent study, however, found that NF-positive nerve fiber density is increased in adenomyotic lesions as compared with controls [43]. Hence whether or not sensory nerve-derived SP and CGRP play any role in lesional progression in adenomyosis remains unclear. Similarly, whether adrenergic receptors or nicotinic AChRs play any role in lesional progression is completely unknown.

Surprisingly, however, the role of receptors for neuropeptides/neurotransmitters secreted by sensory, sympathetic and vegal nerves in adenomyotic lesions has, to our best knowledge, never been investigated. This is unfortunate, since, first, the endometrial-myometrial interface (EMI), which is known to play a role in adenomyosis [58], is richly innervated [59]. In addition, neuropeptides such as SP and CGRP and their receptors have recently been shown to play a promotional role in endometriosis progression and fibrogenesis [44, 45], a feature also shared by adenomyosis [14, 55]. The promotional role of adrenergic signaling also has been implicated in endometriosis [46, 60]. Moreover, the potential of α7nAChR agonists as therapeutics has been shown [54], which provides a cue that the AChR signaling pathway or, more broadly, the vagal activity, or lack thereof, may play some roles in adenomyotic progression and fibrogenesis.

In essence, this study provides, for the first time, a survey of the expression patterns and levels of SP receptor NK1R, CGRP receptors CRLR and RAMP1, adrenergic receptor ADRB2, and acetylcholine receptor α7nAChR in adenomyotic lesions. We found that the staining levels of CRLR, RAMP1, NK1R and ADRB2 are all significantly elevated in adenomyotic lesions as compared with control endometrium. In contrast, α7nAChR staining levels were significantly reduced. One notable result is the positive correlation between the severity of dysmenorrhea and lesional ADRB2 staining levels. This suggests that, similar to endometriosis [47], there may also exist a similar feed-forward loop in adenomyosis.

As adenomyotic and endometriotic lesions are both ectopic endometrium and share the same hallmark of cyclic bleeding [12], our results suggest that, as in endometriosis, sensory nerve-derived neuropeptides such as SP and CGRP and sympathetic nerve-derived neurotransmitters such as noradrenaline may be actively involved in the promotion of adenomyosis progression through their respective receptors on adenomyotic lesions. In contrast, vagus nerve derived neurotransmitter acetylcholine might stall the progression. Additionally, through the activation of the hypothalamic-pituitary-adrenal (HPA)-sympatho-adrenal-medullary (SAM) axes and the lesional overexpression of ADRB2, adenomyosis-associated dysmenorrhea and adenomyotic lesions may be mutually promotional, forming a viscous feed-forward cycle.

Our findings of lesional overexpression of NK1R and CRLR/RAMP1 are broadly consistent with the documented roles of SP/CGRP and their receptors in wound healing and fibrogenesis. SP is known to facilitate the normal acute and chronic wound healing processes [24–26]. In contrast, sensory denervation impairs cutaneous wound healing through increased apoptosis and reduced proliferation [27, 28]. In addition, our findings are in agreement with the report that NK1R is expressed in endometriotic lesions, especially in peritoneal lesions [61]. SP enhances, while NK1R antagonism reduces, endometrial stromal cell viability, and treatment of endometrial cells with TNFα induces NK1R expression [61]. Increased nerve fiber density in adenomyotic myometrium [41, 43] may result in elevated SP/CGRP concentration within lesions.

Despite the fact that this study provides, to our best knowledge, the first survey of the some important neuropeptide and neurotransmitter receptors in adenomyotic lesions, our study has several limitations. The most conspicuousl one is that our study is limited by the use of histologic and immunohistochemistry analyses only and lacks molecular data. In addition, we did not evaluate the role of receptors for other neuropeptides such as vasoactive intestinal peptide (VIP) since sensory nerves also secrete neuropeptides other than SP and CGRP. Along the same line, we did not evaluate other adrenergic receptors, such as ADRB1 and ADRB3, nor did we evaluate other acetylcholine receptors such as muscarinic receptors (mAChRs) and other nicotinic AChRs. Neither did we evaluate receptors for other neurotransmitters secreted by glutamatergic, dopaminergic, serotonergic, and GABAergic neurons. These receptors may play important roles in adenomyosis progression and in causing adenomyosis-related symptomology. For example, loss of GABAergic inhibition in mice with induced adenomyosis may exacerbate pain [62]. Similarly, dopaminergic signaling may also be crucial in the prevention or hindrance of adenomyosis [63, 64], especially in view of the evidence that dopamine D2 receptor (DRD2) signaling is seemingly depressed in the development of endometriosis [65–67]. Future studies are needed to elucidate their involvement, if any, in adenomyosis.

Pain, AUB and infertility are three major complaints that prompt women with adenomyosis to seek medical attention. Pain and infertility themselves are known to be potent stressors, causing anxiety and depression [68, 69]. Closely associated with lower quality of life [70], AUB also can induce psychological stress, depression, and anxiety [71, 72]. In particular, adenomyosis-associated pain can be intense and debilitating and typically chronic and uncontrollable, and the psychological stress thus induced appears to contain all the ingredients for exerting a potent negative effect on women with adenomyosis [73]. As a result, it is likely to induce systemic activation of the HPA and the SAM axes, resulting in increased release of glucocorticoids and catecholamines. The catecholamines, especially adrenaline and noradrenaline, would activate the ADRB2/CREB signaling pathway in lesions, inducing angiogenesis and proliferation and leading to accelerated progression of adenomyosis as in endometriosis [46]. The accelerated progression may further exacerbate pain, effectively forming a vicious cycle. This may explain as why the lesional ADRB2 staining was associated with the severity of dysmenorrhea.

## Conclusions

We found increased lesional staining levels of CRLR, RAMP1, NK1R and ADRB2 but decreased staining levels of α7nAChR in adenomyotic lesions as compared with control endometrium. In particular, the severity of dysmenorrhea correlated positively with the lesional ADRB2 staining levels. Our results suggest that sensory nerve-derived neuropeptides such as SP and CGRP and sympathetic nerve-derived neurotransmitters such as noradrenaline may promote the development of adenomyosis through their respective receptors on adenomyotic lesions. In contrast, vagus nerve derived neurotransmitter acetylcholine may stall the progression of adenomyosis. Our data also suggest that, similar to endometriosis [47], there may also exist a feed-forward loop in adenomyosis. Above all, our results suggest that receptors of neuropeptides and neurotransmitters may play roles in the development of adenomyosis and its related symptomology. However, our study also unveils another layer of complex wrinkles in adenomyosis that are in need of further investigation.

## Supplementary Information

**Additional file 1.**

## Data Availability

The de-identified supporting data are available from the senior author upon written and reasonable request.
