# Supplementary material for: Possible involvement of neuropeptide and neurotransmitter receptors in Adenomyosis
Source: Reprod Biol Endocrinol. 2021 Feb 19;19:25. doi: 10.1186/s12958-021-00711-6 (PMC7893711; doi:10.1186/s12958-021-00711-6)
Supplement: Supplementary file 1 — Additional file 1. [file 12958_2021_711_MOESM1_ESM.docx]

**Possible Involvement of Neuropeptide and Neurotransmitter Receptors in Adenomyosis**

**Xiaofang Xu, Xianjun Cai, Xishi Liu, and Sun-Wei Guo**

**Supplementary Information**


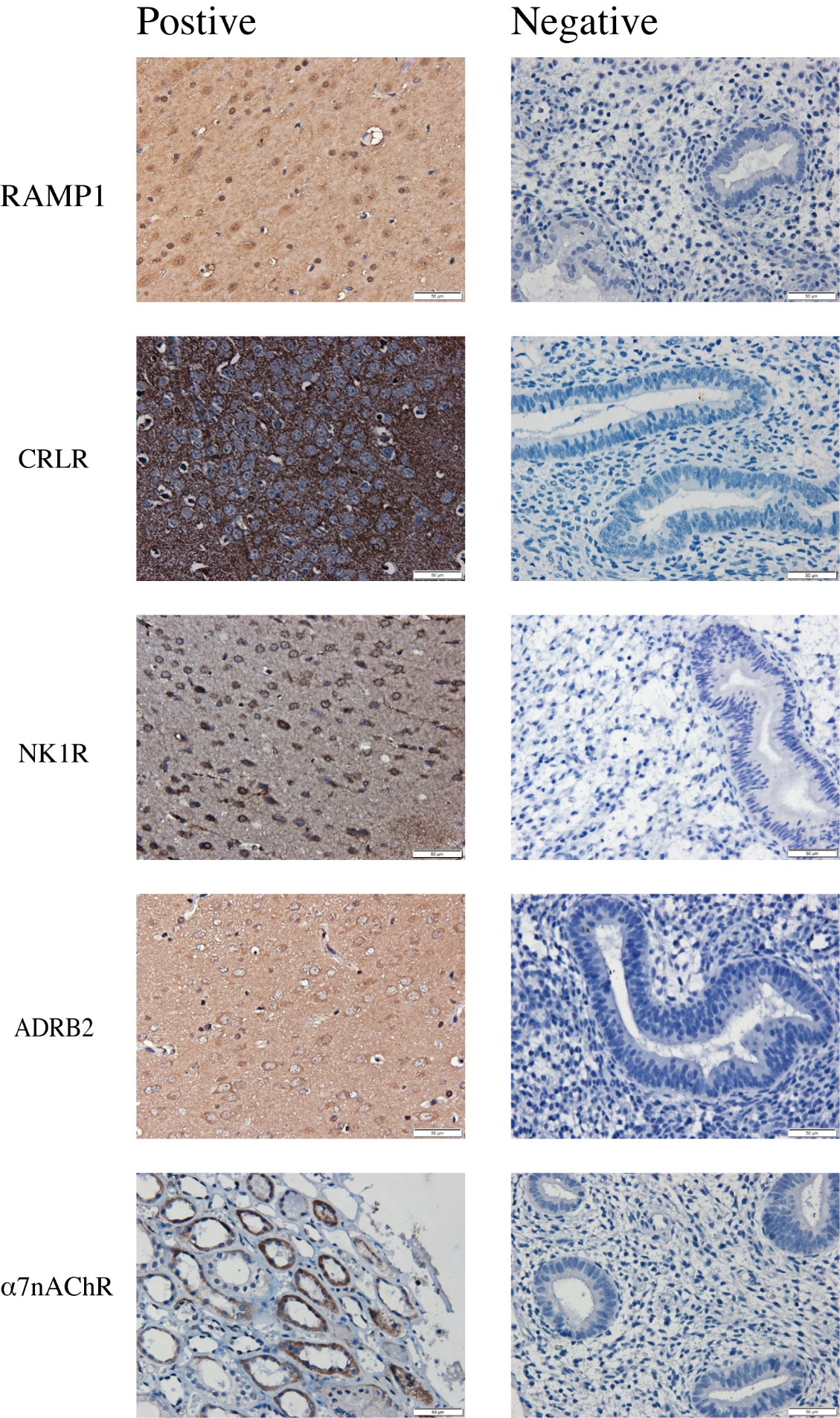


**Figure S1. Positive and negative controls for immunohistochemistry.** Representative immunostaining of RAMP1, CRLR, NK1R, ADRB2 and α7nAChR in mouse brain (RAMP1, CRLR, NK1R, ADRB2) and mouse kidney (α7nAChR) tissue samples (left column) shown as positive controls. The negative controls were stained in human adenomyosis (RAMP1, CRLR, NK1R, and ADRB2) and human endometrium (α7nAChR) tissue samples (right column). Magnification in all figures: X400. The scale bar represents 125 μm.
